# Therapeutic Interchange of Parenteral Anticoagulants: Challenges for Pharmacy and Therapeutics Committees

**DOI:** 10.3390/ph4111475

**Published:** 2011-11-07

**Authors:** Alpesh Amin

**Affiliations:** University of California-Irvine, 101 The City Drive South, Building 26, Room 1005, ZC-4076H, Orange, CA 92868, USA; E-Mail: anamin@uci.edu; Tel.: +1-714-456-3785; Fax: +1-714-456-3875

**Keywords:** therapeutic interchange, anticoagulant, venous thromboembolism

## Abstract

This is a review of key factors for pharmacy and therapeutics committees to consider when developing a therapeutic interchange (TI) program for venous thromboembolism (VTE) prophylaxis. Recent patient safety initiatives aimed at reducing the incidence of hospital-acquired VTE may increase the prescribing of thromboprophylactic agents recommended in VTE management guidelines. As a result, more pharmacy and therapeutics committees may consider TI programs for parenteral anticoagulants. However, the TI of anticoagulants appears challenging at this time. Firstly, the therapeutic equivalence of the commonly prescribed parenteral anticoagulants, enoxaparin, dalteparin and fondaparinux, has not been established. Secondly, because of the wide range of clinical indications for these anticoagulants, a blanket agent-specific TI program could lead to off-label use. Use of an indication-specific TI program could be difficult to manage administratively, and may cause prescribing confusion and errors. Thirdly, careful dosing and contraindications of certain parenteral anticoagulants in special patient populations, such as those with renal impairment, further impact the suitability of these agents for inclusion in TI programs. Finally, although TI may appear to offer lower drug-acquisition costs, it is important to determine its effect on all cost parameters and ultimately ensure that the care of patients requiring VTE prophylaxis is not compromised.

## 1. Introduction

Venous thromboembolism (VTE) is a major cause of preventable mortality and morbidity in hospitalized patients, and is associated with substantial clinical and economic burden [1,2]. Effective pharmacologic methods with good safety profiles are available to prevent VTE [1]. However, VTE prophylaxis is widely underutilized [3-6], and effective protocols are needed to minimize the incidence of hospital-acquired VTE.

Patient safety initiatives aimed at improving practice have already been introduced [7,8], with performance measures increasingly being incorporated into public reporting initiatives, incentive schemes, and pay-per-performance programs [9,10]. With the growing recognition of this major public health problem, a significant increase in the use of parenteral anticoagulants for VTE prophylaxis may be expected, and more hospitals appear to be incorporating them into their therapeutic interchange (TI) programs [11,12]. TI is defined as the dispensing of a drug that is therapeutically equivalent to, but chemically different from the drug originally prescribed by a physician or other authorized prescriber [13]. The main goal of TI programs is to reduce the total cost of therapy and help develop standardization of usage within an institution, while not compromising patient care [13]. This article outlines some of the key factors that need to be considered when developing TI programs, using the case example of VTE prophylaxis and parenteral anticoagulants.

## 2. Agents for VTE Prophylaxis

Anticoagulants recommended by evidence-based VTE management guidelines include orally administered warfarin, as well as parenteral unfractionated heparin (UFH), low-molecular-weight heparins (LMWHs), and fondaparinux (Arixtra^®^-GSK) [1,14]. Available LMWHs in the U.S. are enoxaparin (Lovenox^®^-sanofi-aventis), dalteparin (Fragmin^®^-Eisai Inc.-Pfizer), and tinzaparin (Innohep^®^-Leo Pharma). There are currently many novel anticoagulants in various stages of phase II, III, and IV trials, but our focus will be on the TI issues for the existing parenteral anticoagulants.

Biosimilar LMWHs, which have recently been approved by the FDA, are an additional consideration [15]. LMWHs are produced through complex depolymerization processes, which cannot be exactly replicated in the production of a biosimilar formulation. Therefore, equivalence of the biosimilar product cannot be ensured [16]. The FDA have based their approval on demonstration of sameness according to five criteria, which involve (1) the physical and chemical characteristics of the specific LMWH; (2) the nature of the heparin material and the chemical process used to break up heparin chains into smaller pieces; (3) the nature and arrangement of components that constitute the LMWH; (4) biological and biochemical assays on the product's anticoagulant activity; and (5) *in vivo* pharmacodynamic profile. As the biosimilar LMWHs have only recently been produced there is limited clinical data available with which to compare the biosimilar and branded versions.

## 3. Considerations for TI Programs

In line with recommendations from the American College of Clinical Pharmacy, several steps have been suggested for consideration by Pharmacy and Therapeutics (P&T) committees when evaluating if parenteral anticoagulants are suitable for inclusion in a TI program (Table 1) [17]. Based on the factors discussed below, the TI of parenteral anticoagulants appears challenging at this time.

### 3.1. Equivalence of Efficacy and Safety

A key consideration relates to determination of clinical equivalence. LMWHs are a heterogeneous mix of polysaccharide chains of differing lengths and weights uniquely prepared from UFH. Although their anticoagulant properties are similar, each LMWH is a distinct pharmacologic entity. As such, LMWHs have different biochemical, biophysical, and pharmacologic properties that affect their clinical efficacy and safety [18].

The LMWHs and fondaparinux are also chemically and pharmacologically distinct agents. The major pharmacokinetic differences between LMWH, UFH, and fondaparinux are listed in Table 2 [19-21].

Fondaparinux is a pentasaccharide that selectively modulates one step in the tissue factor pathway by inactivating factor Xa; in contrast, LMWHs inhibit multiple steps in the tissue factor pathway and also weakly inhibit the contact activation pathway [22].

To be appropriate for TI, parenteral anticoagulants must be therapeutically equivalent both in clinical trials and real-life settings [13]. Clinical evidence demonstrating equivalent therapeutic efficacy of the different LMWH agents and fondaparinux in a blanket fashion is lacking. In a head-to-head comparison between fondaparinux (2.5 mg once daily) and enoxaparin (30 mg twice daily) after major knee surgery, fondaparinux was associated with a lower incidence of symptomatic or asymptomatic VTE (12.5% *versus* 27.8%; p < 0.001), but a higher incidence of major bleeding (2.1% *versus* 0.2%; p = 0.006) [23]. Studies that have compared a LMWH or fondaparinux with placebo or UFH have had varying study designs and endpoints, making comparison of results difficult. In summary, therapeutic equivalence of enoxaparin, dalteparin, tinzaparin, and fondaparinux are challenging to establish at present because of limited head-to-head comparisons, reports of differences in the efficacy and safety profiles in certain patient populations, and unequal distribution of clinical evidence.

### 3.2. Indications

It is also important to compare the indications of the drugs under consideration for TI. This is of particular relevance during a blanket TI program in which one agent is dispensed for another for various indications rather than an indication-specific interchange. The Food and Drug Administration (FDA)-licensed indications for enoxaparin, dalteparin, tinzaparin, and fondaparinux extend beyond VTE prophylaxis to VTE treatment and acute coronary syndromes (ACS) Table 3 [21,24-26].

Enoxaparin has the largest number of FDA-approved indications compared with the other parenteral anticoagulants. Blanket interchange of one agent for another would lead to off-label use. Although physicians in the U.S. are not restricted from prescribing agents for off-label use, P&T committees should ensure that interchange does not negatively impact outcomes.

Authoritative bodies such as the American College of Chest Physicians (ACCP) and American Heart Association/American College of Cardiology (AHA/ACC) have produced guidelines on VTE management and ACS care, which can provide guidance on the suitability of the different parenteral anticoagulants in these settings (Table 4) [1,27-29]. For ACS, the AHA/ACC guidelines state that UFH, enoxaparin, and fondaparinux each satisfy criteria for effectiveness, but it is often difficult to conclude that one antithrombotic strategy is preferred over another [28,29]. For VTE indications, evidence-based guidelines from the ACCP recommend that the choice between a LMWH or fondaparinux should be based on the clinical indication, the level of evidence for each agent in that indication, and the approval of the regulatory bodies [1,14,27]. They do not provide specific recommendations for the different LMWHs other than referring back to the individual agents' product specifications for dosing [1,27].

Indication-specific TI can take the form of TI in a single indication, for example replacing enoxaparin with dalteparin in VTE prevention after knee replacement [30]. However, dalteparin does not have an indication for VTE prevention after knee replacement. Alternatively, replacement of one agent with two other agents, has been considered in some hospitals to cover more indications. In such a dual TI program, enoxaparin has been replaced with dalteparin and fondaparinux for each of their approved VTE indications [31].

Indication-specific programs can be complicated to manage administratively, may incur additional costs, and may need educational initiatives. The need for additional measures and education as a result of a TI program may act as a barrier to improved compliance with VTE prophylaxis recommendations. This may make it difficult to ensure that an organization achieves the outcomes required by regulatory organizations for patient safety, quality, certification, and payment. In addition, indication-specific TI with one or more than one agent may cause prescribing confusion and potentially increase the opportunity for errors [17]. P&T committees should consider all these factors when reviewing the need for TI.

### 3.3. Special Populations

The suitability of drugs for TI may also depend on the patient populations involved. Special considerations may be necessary if the patient population contains large numbers of elderly, obese or pregnant patients, or if patients frequently have other complicating factors such as comorbidities. Effects on humanistic variables, including quality of life, functional status, and patient satisfaction should also be considered [13].

Patients who are elderly, obese, of very low body weight, or have renal impairment require careful dosing of anticoagulants, and dose adjustment may be needed as detailed in Table 5 [21,24-26]. FDA-approved dose regimens are available for enoxaparin for elderly patients and patients with severe renal impairment (creatinine clearance [CrCl] <30 mL per min) [24]. Alternatively, dalteparin does not have specific dose regimens for patients with severe renal impairment; however, a maximum dose cap is recommended in obese patients treated for non-ST-segment elevation ACS and patients with cancer receiving extended VTE treatment. For tinzaparin a warning has been issued by the manufacturer to consider alternatives to tinzaparin for treatment of VTE in elderly patients with renal insufficiency (http://www.innohepusa.com/), based on interim data from a clinical study (IRIS; Innohep in Renal Insufficiency Study). As mentioned, fondaparinux is contraindicated in patients with severe renal impairment (CrCl <30 mL per min) and as VTE prophylaxis in patients weighing less than 50 kg [21]. These significant differences in the indications for usage of various parenteral anticoagulants in special populations can make TI quite challenging.

### 3.4. Costs

In the current literature, there are no pharmacoeconomic analyses regarding interchanging parenteral anticoagulants. A few studies have investigated the economic consequences of a TI program changing from enoxaparin to dalteparin [30,32-35]. In 1996, the P&T committee of the University of Wisconsin Hospital and Clinics initiated a TI program in which enoxaparin was replaced with dalteparin as the only LMWH on formulary [32]. An annual reduction in LMWH acquisition costs of approximately $90,000 was reported. Observed rates of VTE and bleeding complications with dalteparin (n = 90) after TI were reported to be similar to a literature control group of enoxaparin (n = 5,578) [32].

A retrospective cohort study of 461 patients assessed a formulary switch from enoxaparin to dalteparin as prophylaxis for deep-vein thrombosis (DVT) in patients undergoing inpatient rehabilitation following total hip arthroplasty or total knee arthroplasty [35]. Adjusted *per capita* costs for DVT prophylaxis drug-acquisition and dispensing were $129 lower among patients treated with dalteparin, and the authors noted that patient care did not appear to be compromised. In a study from the Franciscan Health System, costs associated with inpatient health care use (which included costs such as room and board, and drug costs) were found to be equivalent when patients were treated with either enoxaparin or dalteparin for total knee replacement surgery [33]. Another study evaluating the TI of enoxaparin to dalteparin after total knee replacement in 40 patients reported small increases in drug expenditure following the switch, compared with large rises in drug costs for VTE prior to the TI [30].

Several studies have suggested that the TI was associated with compromised efficacy [34,36]. A retrospective analysis of symptomatic VTE events suggested compromised efficacy after TI from enoxaparin to dalteparin in 310 patients who underwent orthopedic surgery. The type of LMWH used independently predicted the occurrence of symptomatic VTE, with enoxaparin being more protective than dalteparin (odds ratio 0.39; 95% confidence interval [CI] 0.20–0.80) [36].

A Canadian study investigated the TI of enoxaparin to dalteparin for VTE prophylaxis in 135 patients with acute spinal cord injury and/or major orthopedic trauma; again, compromised efficacy was suggested [34]. Symptomatic DVT or pulmonary embolism was reported in one patient who received enoxaparin (1.6%) and seven patients who received dalteparin (9.7%), although the difference was not statistically significant (p = 0.103; absolute risk 8.1%; 95% CI −0.6 to 15.6). No differences were observed between enoxaparin and dalteparin when major bleeding (6.4% *versus* 6.9%, respectively; p = not significant) and mortality (4.8% *versus* 6.9%; p = 0.865) were assessed. Switching from enoxaparin to dalteparin was associated with savings of $12,485 Canadian dollars in LMWH acquisition costs over the 1-year study period. Despite these savings in acquisition costs, the authors concluded that enoxaparin should still be the prophylactic agent of choice because their data suggested compromised efficacy with dalteparin with regard to symptomatic DVT [34].

An important limitation of the majority of published TI studies is that acquisition costs alone were analyzed and this may not reflect savings in overall hospital expenditure. P&T committees need to consider the effect of TI on total expenditure, including the substantial costs of managing any negative outcomes, such as additional VTE events or adverse events. In addition to the cost of managing negative clinical outcomes, it is important to consider the staff resources required to set up and implement the program, and the cost of initiating TI [13]. TI may also add time to the dispensing process for pharmacists because of the steps involved in order to make a change to a prescription and, in some cases, the necessary patient education.

### 3.5. Views of Regulatory Organizations and Medical Associations

The views of regulatory organizations and medical associations may be helpful in assessing the suitability of specific parenteral anticoagulants for TI. Due to the uniqueness of each agent and the lack of evidence supporting therapeutic equivalence, the World Health Organization, the International Union of Angiology and the South Asian Society of Atherosclerosis and Thrombosis, as well as U.S. national organizations, including the ACCP, AHA/ACC and The North American Thrombosis Forum, state that the LMWHs are distinct, non-interchangeable agents [18,20,28,37].

## 4. Conclusions

As a result of the expansion in the number of drugs within the same or comparable therapeutic classes, and the need to control drug and related health care expenditure, TI is increasingly being considered by P&T committees as a method of managing these issues in the hospital pharmacy. Current patient safety initiatives aimed at reducing the burden of VTE will lead to an increase in the prescribing of thromboprophylaxis and could lead to more hospitals considering parenteral anticoagulants for a TI program. A key criterion for assessing the suitability of any agent for inclusion into a TI program, relates to the therapeutic equivalence of the agents in question. However, the therapeutic equivalence of the commonly prescribed parenteral anticoagulants enoxaparin, dalteparin, and fondaparinux in a blanket fashion is not supported by head-to-head trials and they are not regarded by regulatory authorities as interchangeable agents. The approach of an indication-specific TI program is more difficult to manage than a blanket TI for an agent, which could lead to increased opportunity for errors. A TI program for parenteral anticoagulants is also complicated by considerations related to their use in special populations, such as the elderly, the obese and those with renal impairment, who require careful dosing and specific dose adjustments. Although a TI program may be associated with lower drug-acquisition costs, it is important to determine its effect on all cost parameters, including staff resources to design, implement and audit the program, and the provision of additional dispensing time.

An inappropriate TI program that fails to fully consider clinical and humanistic outcomes of care may eventually result in increased hospital costs. For example, reduced efficacy and safety may lead to further VTE or bleeding complications, and poor patient satisfaction may reduce patient compliance and, therefore, drug effectiveness. P&T committees should not be driven by drug-acquisition cost as the sole motivation for TI. Rather, committees should look at whether the available evidence supports a TI in terms of therapeutic equivalence, patient safety, and practical considerations.

## Figures and Tables

**Table 1.** Steps for pharmacy and therapeutics committees evaluating an anticoagulant TI program.

○ Review all published clinical evidence to assess the therapeutic equivalence of agents in the interchange ○ Analyze the clinical efficacy and safety data for each VTE indication and other clinical situations, such as pregnancy, neurosurgery, pediatric surgery, and perioperative management ○ Assess Food and Drug Administration-licensed indications of each agent, allowing an evaluation of the extent of off-label use involved in the interchange ○ Consider the impact of the TI on patient populations with specific anticoagulant dosing requirements (e.g., elderly, renally impaired, obese, low weight) ○ Evaluate the pharmacoeconomic implications of the switch, considering the total cost of care associated with the interchange, not just the cost of drug acquisition ○ Incorporate an opportunity for physicians to override the TI policy and prescribe a specific anticoagulant when required ○ Plan the education of prescribers/healthcare professionals concerning the TI ○ Implement regular monitoring of the effect of the TI on VTE outcomes (e.g., VTE incidence/recurrence, bleeding complications, costs)

Adapted from Gainor *et al.* [17].

**Table 2.** Pharmacologic properties of UFH, enoxaparin, dalteparin, tinzaparin, and fondaparinux [19-21].

	**UFH**	**Enoxaparin**	**Dalteparin**	**Tinzaparin**	**Fondaparinux**
Manufacturing process	Biological extraction	Benzylation followed by alkaline hydrolysis	Controlled nitrous acid depolymerization	Enzymatic depolymerization	Synthetic
Mean molecular weight, Da	3,000–30,000	4,500	6,000	6,500	1,728
Elimination half-life, hours	Dose-dependent	4.5	3–5	3.9	17
Bioavailability, %	15–25	90–92	87	87	100
Anti-Xa:IIa ratio	1	3.8	2.7	2.8	Anti-Xa selective
Anti-Xa activity, IU/mg	193	100	156	100	700
Neutralization	Protamine sulfate	Protamine sulfate (incomplete)	Protamine sulfate (incomplete)	Protamine sulfate (incomplete)	NA

NA = not applicable.

**Table 3.** Summary of FDA-approved indications for enoxaparin, dalteparin, tinzaparin, and fondaparinux [21,24-26].

**Indication**	**Enoxaparin**	**Dalteparin**	**Tinzaparin**	**Fondaparinux**
VTE prophylaxis				
Hip replacement surgery	Yes	Yes	No	Yes
Knee replacement surgery	Yes	No	No	Yes
Hip fracture surgery	No	No	No	Yes
Abdominal surgery	Yes	Yes	No	Yes
Acutely-ill medical patients	Yes	Yes	No	No
VTE treatment	Yes	No	Yes	Yes
Extended VTE treatment in cancer patients	No	Yes	No	No
Unstable angina/non-ST-segment elevation MI	Yes	Yes	No	No
ST-elevation MI	Yes	No	No	No

MI = myocardial infarction.

**Table 4.** Summary of ACCP [1,27] and AHA/ACC recommendations [28,29] for UFH, the LMWHs, and fondaparinux.

**Indication**	**UFH**	LMWH *			**Fondaparinux**
VTE prophylaxis					
Hip replacement surgery	Against: Grade 1A ^†^	Grade 1A			Grade 1A
Knee replacement surgery	Against: Grade 1A ^†^	Grade 1A			Grade 1A
Hip fracture surgery	Grade 1B	Grade 1B			Grade 1A
General surgery	Grade 1A	Grade 1A			Grade 1A
Acutely ill medical patients	Grade 1A	Grade 1A			Grade 1A
VTE treatment	Grade 1C ^‡^	Grade 1A			Grade 1A
Extended VTE	No	Grade 1A (3 months)			No
treatment in cancer patients		Grade 1C (until cancer resolved)			
**Indication**	**UFH**	**Enoxaparin**	**Dalteparin**	**Tinzaparin**	**Fondaparinux**
Unstable angina/non-ST-segment elevation MI					
Invasive strategy	Class 1 (Level of evidence A)	Class 1 (Level of evidence A)	No	No	Class 1 (Level of evidence B)
Conservative strategy	Class 1 (Level of evidence A) ^§^	Class 1 (Level of evidence A)	No	No	Class 1 (Level of evidence B)
ST-elevation MI	Class 1 (Level of evidence C) ^¶^	Class 1 (Level of evidence A)	No	No	Class 1 (Level of evidence B)

* For recommendations on the dosing of specific LMWHs, the ACCP refers to the individual product specifications.

† Recommendations against use as the sole prophylactic agent.

‡ SC LMWH recommended over IV UFH for the initial treatment of acute deep-vein thrombosis, except for patients with severe renal failure (UFH recommended); SC LMWH recommended over IV UFH for the initial treatment of acute non-massive pulmonary embolism, except for patients with severe renal failure or if there is concern about SC absorption or thrombolytic therapy is planned (IV UFH recommended).

§ Enoxaparin or fondaparinux preferred over UFH unless coronary artery-bypass graft is planned within 24 hours.

¶ Regimens other than UFH recommended if anticoagulant therapy is given for more than 48 hours. IV = intravenous; SC = subcutaneous.

**Table 5.** Dosage considerations for parenteral anticoagulants in special patient populations [21,24-26].

**Drug**	**Renal impairment**	**Obese patients**	**Patients with low body weight**	**Elderly patients (≥ 75 years)**
Enoxaparin	FDA-approved dose adjustment in patients with CrCl <30 mL per min	Dose cap: - 100 mg for first two SC doses only in ST-elevation MI patients (≥ 100 kg)	Observe for signs of bleeding	FDA-approved dose adjustment in ST-elevation MI patients
Dalteparin	Patients with CrCl <30 mL per min should be monitored for anti-Xa levels to determine the appropriate dose	Dose cap: - 10,000 IU per dose in non-ST-elevation MI patients (≥ 83 kg) - 18,000 IU per day in extended VTE treatment in cancer patients (≥ 83 kg)		
Tinzaparin	Consider the use of alternatives in elderly patients with renal insufficiency	Weight-based dosing is appropriate for heavy/obese patients - 175 IU/kg or 75 IU/kg		Consider the use of alternatives in elderly patients with renal insufficiency
Fondaparinux	Contraindication: patients with CrCl <30 mL per min	Dosing in VTE treatment: - 5.0 mg for body weight <50 kg - 7.5 mg for 50–100 kg - 10.0 mg for >100 kg	Contraindication: VTE prophylaxis in patients <50 kg	
